# Beyond the School Walls: Keeping Interactive Learning Environments Alive in Confinement for Students in Special Education

**DOI:** 10.3389/fpsyg.2021.662646

**Published:** 2021-04-08

**Authors:** Garazi Álvarez-Guerrero, Ane López de Aguileta, Sandra Racionero-Plaza, Lirio Gissela Flores-Moncada

**Affiliations:** ^1^Faculty of Psychology and Education, University of Deusto, Bilbao, Spain; ^2^Department of Sociology, University of Barcelona, Barcelona, Spain

**Keywords:** special education needs, dialogic gatherings, interactive learning environments, successful educational actions, distance learning

## Abstract

The COVID-19 pandemic and the accompanying safety measures, including confinement, has meant an unprecedented challenge for the world population today. However, it has entailed additional difficulties for specific populations, including children and people with disabilities. Being out of school for months has reduced the learning opportunities for many children, such as those with less academic resources at home or with poorer technological connectivity. For students with disabilities, it has entailed losing the quality of the special attention they often need, in addition to a more limited understanding of the situation. In this context, a case study was conducted in a special education classroom of a secondary education school. This class started implementing Dialogic Literary Gatherings with their special education students before the COVID-19 confinement and continued online during the confinement. Qualitative data was collected after a period of implementation of the gatherings showing positive impacts on the participants. The case study shows that interactive learning environments such as the Dialogic Literary Gatherings can provide quality distance learning for students with disabilities, contributing to overcome some of the barriers that the pandemic context creates for the education of these students.

## Introduction

The pandemic caused by COVID-19 has led most countries to take measures in order to stop the disease from spreading. One of the most effective measures is social distancing, and thus, school closure has been a measure implemented in many countries to prevent new infections (Enserink and Kupferschmidt, 2020). Closing schools has affected more than 1,300,000,000 learners worldwide (Unesco, 2020). However, members from vulnerable groups (individuals with poor financial resources, poor health literacy, or with self-reported disabilities) have faced a greater adversity in relation to mental health, especially anxiety and depression (Reading Turchioe et al., 2021), and access to education (Long et al., 2020). This is the case of individuals with intellectual disabilities, who have suffered greater consequences during the pandemic (Courtenay, 2020). Although people with intellectual disabilities are conceived as a heterogeneous collective, research has found that having a cognitive impairment entails additional challenges facing the COVID-19 situation. On the one hand, taking into account that some online content (such as websites or mobile content) is inaccessible for people with cognitive disabilities, some essential information posted by health authorities about the COVID-19 has remained out of reach (Dror et al., 2020). On the other hand, most measures taken for the continuity of education have been aimed at mainstream education, whereas many individuals with disabilities have not had access to special education during the pandemic (Mutluer et al., 2020).

### COVID-19 Impact on Individuals With Intellectual Disabilities

Although the pandemic caused by COVID-19 has had a negative impact on the global population, people with disabilities have faced many additional challenges. In terms of health, they did not receive enough attention regarding their access to healthcare during the pandemic, due to the new barriers telemedicine has supposed for people with disabilities (Annaswamy et al., 2020). Even research (Wilson, 2020) has shown that the human rights of people with mental and cognitive impairments have been neglected, as some governments' emergency plans have not included their special needs. As an addition to the already existing vulnerability, in relation to the dependence of certain services and other people, the pandemic has brought new challenges to individuals with intellectual disabilities, such as the limited access to information on the disease or its understandability, the risk of losing home support, increasing distress, and behavioral problems (Courtenay, 2020).

In addition, the mental health of persons with intellectual disabilities can deteriorate, as the sudden changes of their routines and the possible obsession with information about COVID-19 may trigger anxiety and paranoia (Courtenay and Perera, 2020). Moreover, social isolation can cause a higher difficulty to access social and emotional support to deal with the grief and stress the pandemic has caused (Lund et al., 2020). Besides, research has emphasized the need of psychological support due to the mental health problems the pandemic may have triggered (Mukhtar, 2021).

Among the many challenges people with intellectual disabilities have faced during the pandemic, those related to the support structures have been pointed out (Courtenay, 2020; Embregts et al., 2020). For young children and children with disabilities and other special needs, the new reality created by the measures to stop COVID-19 from spreading (such as social distancing) may cause anxiety, frustration, and negative behaviors, as for many of these children, expressing their emotions may be difficult (Kong and Thompson, 2020). Thus, scientific literature has stressed out the importance of these children staying connected with their social support system, such as family members, caregivers, teachers, therapists, and friends. In addition, the situation has affected not only children with intellectual disabilities but also their families who suffered high levels of stress, associated with isolation, illness, and finance (Manning et al., 2020).

### Impact of School Closure on Students With Special Educational Needs

School closure has aroused serious challenges for students, teachers, and families, as the shift toward an online education has not been easy to adapt for any of them (Cen et al., 2020; Kim and Asbury, 2020). However, those students with special educational needs (SEN) and their families are facing even greater problems. Difficulties to balance working from home while taking care of their children with disabilities have been pointed out (Hole and Stainton, 2020), as well as the loss of essential resources such as educators or structured learning environments (Masonbrink and Hurley, 2020). In addition, research done before the pandemic had shown the benefits interactive learning environments have on students with special needs, which improve the quality of the education these students are usually given (García-Carrión et al., 2018; Fernandez-Villardon et al., 2020). However, school closure and social distance may put in jeopardy these interactive environments that have been considered so important in special education.

In order to support people with intellectual disabilities during the pandemic, it has been emphasized that the measures taken should focus not only on the prevention of contracting the disease but should also aim to mitigate the effects some of those measures have on these individuals (Constantino et al., 2020). Regarding children, it should be taken into consideration that special education usually involves visual and physical contact, close attention, and interpersonal encouragement. In this vein, evidence has indicated that, while parents have reported to have received guidance from their children's schools, dissatisfaction has been expressed regarding the appropriateness of the resources given, as some of them perceived those resources did not meet their children's educational and psychological needs (Greenway and Eaton-Thomas, 2020). Therefore, many parents have expressed to feel unprepared to provide their children with the appropriate education during school closure, and school closure has led to higher levels of stress on caregivers, for instance, in the case of students with autism spectrum disorder (Manning et al., 2020). Furthermore, many educational interventions provided by governments have not taken into account the rights and voices of persons with disabilities in relation to the digital divide that affects their access to education (Toquero, 2020).

Maintaining contact with the school during the pandemic has been an advice given by researchers in the field (Narzisi, 2020), suggesting that, at least, weekly contact with one of the class companions should be made, as well as with teachers. Parent–teacher collaboration and communication during remote teaching has proven to be very important (Frederick et al., 2020; Schuck and Lambert, 2020), and, as well as students, parents should receive continuous support by teachers (Stenhoff et al., 2020). In case of students with SEN, research made before the pandemic had already emphasized the importance of parent involvement in the education of their children (Staples and Diliberto, 2010).

In order to ensure educational rights for students with disabilities, strategies must include understanding the student's and family's needs, guaranteeing partnership between schools, families, and communities. Furthermore, it is essential making decisions based on data, promoting ethical evaluation in online environments, and ensuring research-based strategies (Jameson et al., 2020). In addition, the need for teachers to use research-based strategies to ensure family and student engagement has been highlighted (Stenhoff et al., 2020).

Besides the academic drawbacks, school closure and lockdown have led to an increase of child abuse and gender violence (Evans et al., 2020; Lee et al., 2021; Masilamani et al., 2021). Unfortunately, individuals with intellectual disability are especially vulnerable in this vein (Courtenay and Perera, 2020), and girls and women with disabilities are at a higher risk of suffering from gender violence (Samaila et al., 2020). In this context, open doors actions (Roca et al., 2020), consisting of actions based on supportive relationships and a safe environment to avoid child abuse during confinement, have been implemented in many schools, including special education schools. Dialogic Gatherings (Ruiz-Eugenio et al., 2020) are one of these actions, which could prevent abuse and at the same time enhance academic learning.

### Dialogic Literary Gatherings and Students With SEN

Dialogic Literary Gatherings (DLG) are one of the Successful Educational Actions (SEAs) identified in the INCLUD-ED research project (Flecha, 2015) as actions that improve students' educational outcomes in diverse contexts and for diverse student populations. In the DLGs, participants read and discuss those universally considered literature masterpieces by authors such as Kafka, Sappho, Shakespeare, and many others (Flecha, 2000). Now being implemented in more than 6,000 schools from Europe and South America, DLGs have shown to foster profound transformations and critical reflections through sharing the ideas on the texts (López de Aguileta et al., 2020). In addition, egalitarian dialogue is one of the bases of the DLG. Thus, the moderator ensures that all opinions are taken into account and fosters the participation of all regardless of their individual characteristics (Llopis et al., 2016).

Research has shown that educational interventions based on interaction and dialogue between children can have a positive impact on the social skills of students with SEN (Fernandez-Villardon et al., 2020). Besides, even if special schools' students have often received an education based on poor interaction and low expectations, the possibility of creating interactive learning environments in these schools has been evidenced (García-Carrión et al., 2018), contributing to new learning opportunities. Families' role in such environments has also been emphasized as key, as it may help improve the learning of students, especially those with more difficulties (Puigdellívol et al., 2017), and enables an educational support with opportunities of inclusion.

When implemented in regular schools, students with SEN participate in the DLGs in an equal way, minimizing the differences between them and the rest of the students (Molina Roldán, 2015). Research (García-Carrión et al., 2020) has indicated that, through the DLGs, the low expectations and prejudices some students with disabilities have to face can be transformed. In this regard, DLGs have shown not only to contribute to the integration of students with SEN to their class but also to foster their instrumental learning (Molina Roldán, 2015).

Despite the school closure, many schools have continued implementing DLGs online. Research in this vein has revealed that DLGs have fostered profound reflections on students during the lockdown, such as the importance of supportive relationships and friendship (Elboj-Saso et al., 2021). In fact, many schools have transferred the DLG to online spaces. A study by Ruiz-Eugenio et al. (2020) has pointed out that doing DLGs during school closure has promoted children well-being in terms of emotional, educational, and social wellness and reduction of anxiety. In addition, one of the schools from that study was a special school, and results showed that students with special needs have not only improved their linguistic production but have also had feelings of safety thanks to the DLGs.

Moreover, collaboration with social workers via videoconference has proven to be essential for ensuring the well-being of some individuals with disabilities during the pandemic (Redondo-Sama et al., 2020b).

In this case, a double objective is being pursued. The objective of the study is, firstly, to understand how DLGs can be transferred to online learning with students with SEN and, secondly, to analyze the impact online DLGs have had on these students during the lockdown and school closures.

## Case Study

A case study has been developed in a special needs classroom of a high school in the Basque Country, Spain. This class is formed by five students (two girls and three boys) aged between 13 and 21 years old with diverse profiles including educational needs due to a moderate or severe intellectual disability and/or a pervasive developmental disorder. The oral language and literacy abilities of participants are affected, but they all are able to communicate verbally.

In the framework of the research project *INTER-ACT. Interactive Learning Environments for the Inclusion of students with and without disabilities: improving learning, development and relationships* (García-Carrión, 2018-2021), this classroom started to implement DLG with the special education students, taking the compromise of carrying the action out according to the orientation that scientific research has stated for its good functioning. Since the project started, the DLGs were done once a week for 6 months and the adapted version of *Don Quixote* by Cervantes was read and discussed. At this moment, COVID-19 confinement started in Spain, and due to school closure, on the third month of having done the DLGs, they were transferred online. The transference to the online modality was an initiative of the classroom teachers in order to not lose the learning context that had been created, and DLGs were done virtually the 2 months that were left of the school year.

## Materials and Methods

The data collection techniques used in this study were semi-structured interviews, communicative observations, and communicative focus groups.

Observations were conducted of three DLG online sessions in order to analyze the functioning, the way the DLGs were adapted to the virtual context, the interactions created, and the strategies used to ensure the inclusion of everyone. Five students and two teachers were present in the DLG sessions. The three sessions were recorded to facilitate the subsequent analysis.

A communicative focus group was held with four students who had participated in the online DLG and the two teachers. The aim of the focus group was to understand the perceptions and feelings of students regarding the online DLGs and how they felt this experience had helped them. The aspects they liked and disliked, how they perceived the relationships created in the DLGs, and the improvements they perceived were searched.

Finally, a joint semi-structured interview with a communicative orientation was made to the two teachers of the class together. This interview was aimed at getting an insight on the teachers' perceptions about strategies used for the DLGs, interactions, relationships between students, and changes in learning outcomes due to the DLGs.

Both the focus group and the interview were done after the three DLG observation sessions in order to discuss and get a better understanding of what was observed, therefore including the communicative dimension to the data collection and analysis.

In this regard, the case study was conducted following the communicative methodology, which entails researchers and participants engage in an egalitarian dialogue where the researcher provides academic knowledge about the topic of the research while participants contribute their daily life vision (Garcia Yeste et al., 2018). This methodology has been identified as being especially appropriate to conduct research with vulnerable groups (Flecha, 2014) and to create knowledge that fosters social transformation (Gómez et al., 2019; Redondo-Sama et al., 2020a) because of the social and political impact that this methodology allows (Gómez et al., 2011).

The communicative focus group and the interview were video and audio recorded and then fully transcribed. The three DLG sessions were video and audio recorded and the most significant excerpts for the purpose of the study were transcribed and coded.

### Data Analysis

For the data analysis, a coding scheme was built. Two main categories were created: characteristics of the implementation of the DLGs with students in special education during confinement and improvements associated with this implementation of the DLG. For each category, other sub-categories were established.

Regarding the characteristics of the implementation of DLGs, sub-categories were strategies and interactions. Strategies included resources, materials, or adaptations (or a lack of them) that were used with students with SEN. Interactions referred to the interactivity of students, their peers, and volunteers or teachers.

Regarding the second category, related to the improvement associated with the DLGs, it was divided into three sub-categories: students' development, curricular learning, and socialization. Development focused on expressive language, attention, and reasoning improvements. Curricular learning is linked to improvement in instrumental areas (such as literacy). Finally, socialization sub-category was defined as the improvement of social relations within the group.

Following the principles of the communicative methodology (Gómez et al., 2011, 2019), all five sub-categories were divided into the exclusionary and transformative dimensions. Exclusionary elements are those barriers faced by some individuals or groups that prevent them from enjoying or participating from certain areas, whereas the transformative dimension includes the elements that help overcome those barriers (see Table 1).

**Table 1:** Coding scheme.

	**Characteristics of the implementation of the DLG**		**Improvements associated with the implementation of the DLG**		
	**Strategies**	**Interactions**	**Development**	**Curricular learning**	**Socialization**
Transformative dimension	1	3	5	7	9
Exclusionary dimension	2	4	6	8	10

*DLG, Dialogic Literary Gatherings*.

The research followed the principles of the Declaration of Helsinki and was approved by the Ethics Committee of the Community of Research on Excellence for All (CREA). Participant teachers and students' parents or legal guardians were provided with the information of the study and signed an informed consent. They were informed of the anonymity, possibility of withdrawal of the study whenever desired, and that the data would only be used for research purposes. In order to preserve participants' anonymity, the two teachers were coded as T1 and T2 and students as S1, S2, S3, S4, and S5.

## Results

The case study has enabled us to elucidate how DLGs can be implemented online with students with special needs and the impact of the online DLGs during confinement in these students. In this section, we present the results of the study showing, in the first place, the characteristics of these DLGs, that is, the strategies used to facilitate students' participation and the interactions that took place between students, families, and teachers. Then, the improvements observed in the students' development, curricular learning, and socialization will be shown. Both the transformative elements as well as the difficulties are presented.

### Strategies That Facilitated Students With Special Needs to Participate in Online DLG

In face-to-face DLGs, some strategies such as audiobook and providing pictures about the story were used. These were strategies to adapt the regular operation of DLG to the characteristics and needs of the students, while keeping the essence of the DLG focused on dialogic learning based on the texts. During confinement, the strategies observed are also related to adaptations based on the necessities of the students, also considering the context of distance learning. First, when the lockdown started, all students could receive not only the written book but also the audiobook that until that moment was given only for particular students but, in the end, was useful for all of them to access the story. Second, during the observations of the DLG sessions, another recurrent strategy was using the camera to show the pictures and drawings of what was happening in the story, so that all students could see it. This strategy was also used when introducing new vocabulary or to show some actions of the sequence of the story. T1 also employed significant gestures to depict what was happening in the story, such as pointing the finger to her head to represent that *Don Quixote* was not in his right mind.

Thus, it was seen that these strategies and adaptations were related to taking advantage of the available technology, in a way that could help overcome the difficulties the lockdown situation presented, mainly by enhancing the possibilities of students' learning, participation, and interaction.

Greater difficulties were perceived for the students' understanding of the books as compared to face-to-face gatherings. Therefore, the strategies used were aimed at overcoming such difficulties. Besides, teachers gave great importance to maintaining interaction as a source of learning during the confinement; thus, they took advantage of the available resources to facilitate everyone's participation.

As a teacher explained, having the audio adaptation of the book helped all students to participate in the DLG, as not all of them had a high level of literacy.

“We achieved the audio adaptation of the book for that particular student, but it has been useful for all students (...) because well, with the reading level they had… (...) Thanks to the reading and the audio it was how we could work.” [Teacher 1 (T1) interview]

When students with special needs have not enough reading abilities to read the text on their own, it can be read by others such as the class teacher or other visual supports to help students' understanding of the text can be used, but lockdown entailed a barrier for this to occur and school closure supposed the strategies that were used during face-to-face classes had to be reconsidered. In fact, finding the appropriate strategies was seen by the teacher as more important in the times of school closure.

Teachers included the audiobook to overcome that barrier and ensure that every student could participate in the DLGs, that is, not only to have access to the content of the story and understand it but also to be able to actively participate in the debate as a result of it.

“The audiobook has helped students that maybe, if we were in class, wouldn't have had that problem, because, you know, in the beginning we adapt it even with pictograms and things about the author, the book, the period (...) but having them confined at home, with a difficult text for them (...) the audio has been for many of them even more important that the reading.” (T1 interview)

The use of the camera and other visual supports during the DLG, such as making drawings related to the story, had also the objective of both facilitating the understanding of the story and facilitating students' expression. When some of the students chose an element of the text that they had considered interesting, teachers asked them to show it to the camera, so everyone could see where the excerpt chosen was located in the text and read it out loud.

“Visual adaptations of the book, a little bit of adventure searching about it on the Internet and making pictures about it as we have done, has made it richer.” (T1 interview)

### Supporting the Transference of Learning Interactions to the Online DLG: The Key Role of Families

DLGs have allowed the creation of an interaction space during the lockdown. During the time DLGs had been done face-to-face, teachers observed good results in their students, and now they had the challenge to transfer this interactive situation in an online environment. The observed interactions were related, on the one hand, with offering opportunities of learning for every student. On the other hand, a diversity and richness of interactions was seen, where students, teachers, and family members took part. In this context, families played a key role in fostering participation and interaction of the students.

Teachers consider that the essence of the interactions in DLG was transferred online and that DLGs online were an alternative to maintain classroom interactions oriented toward improving learning in the virtual space. The fact of having been implementing face-to-face DLG previously created a context that made teachers see the opportunity to create an interactive context online, which otherwise may have been substituted by individual activity, losing the potential of interaction for learning:

“It has been, well, great, great. Because if we hadn't had this excuse or project to do together, we would probably have had individual classes instead of group classes.” (T1 interview)

The quality of the interactions between students has also been maintained. T1 pointed out that during online DLG, interactions were based on respect, listening to each other and learning new ideas from their peers. These reflect the typical interactions from the DLG based on egalitarian dialogue where all voices are heard and valued, which were transferred to the virtual space.

“[It is like] Sometimes I don't agree with the opinion of my classmate, however, there is always respect, and sometimes they provide an idea that can be useful for me.” (T1 focus group)

In addition, interactions with families were acknowledged as a key component that enabled doing DLGs online. According to the teachers, parents of the students not only helped them connect to the video conference but also showed a high level of compromise regarding the DLGs. For instance, in the case of S3, it was her mother who helped her follow the videoconference. She usually spoke to her daughter in a soft voice whenever she got distracted or did not understand anything or helped her find the paragraph she had chosen and to read:

T1: What have you liked the most about this chapter?
Student 3 (S3): I have liked… (silence)
Mother: Here. Read it.
[S3 reads the whole sentence with the help of her mother. The mother whispers the words whenever S3 gets stuck, and S3 says it out loud]
T1: Very well, S3! You have chosen the same paragraph as S1! Very well. (Observation session 1).

The fact that families were already involved with the project (before the lockdown, they helped students to read at home and underline paragraphs to prepare the face-to-face DLGs) facilitated that they knew the dynamics, the benefits of this action for students, and that they took the compromise of helping to do the DLGs online. Families were perceived as crucial so that students could connect and participate to the DLG sessions online, becoming a “bridge” between the school and students.

“In fact, I believe it has meant a greater compromise for parents too. The fact of having a project and saying: ‘hey, we are compromised,’ there is a compromise, and it meant that parents were taking into account the time, the day, the moment, and that they did it.” (T1, interview).

Students agree with this perception. Two of them stated that it was their parents who helped them prepare the reading for the DLG, and it was something they appreciated and made them feel comfortable with the DLGs online.

Interviewer (In): Who has helped you to prepare for the DLGs? Fathers, mothers, grandparents, teachers…
S3: My mother.
(...)
S2: My mother.
In: And have you liked that she has helped you?
S2: Yes.
(...)
Interviewer: And why do you prefer to do it [the DLGs] with the computer? Because you are at home, with your mum…
S3: Because I am at home with my mum. (Focus group)

In this vein, teachers explained that parents have also considered the DLGs to be a positive experience. At the beginning, teachers mentioned that some of the parents were reluctant because they thought the book was too difficult for their children. However, this perception changed through time, and teachers stressed the positive feedback that parents have given them on this action and the great help they provided to prepare for the DLGs. This shows not only that parents have been a valuable resource to develop online DLGs but also that having the opportunity to participate together with their children and the teachers in this activity helps them to get to know better the functioning of the school, value the work done there, as well as increase the expectations that parents have on their children.

T2: They have taken part, I think that with the fact that when we sent the chapter, they read it.
T1: And underlining the phrase, I mean, making a comment on the text, for me it has been…
T2: They haven't gone against it.
T1: No, no, not at all. I think the assessment of the families has been positive. (Interview)

### Improvements in Students' Development of Cognitive Abilities and Learning Attitudes

Teachers reported an improvement in behavior, attention, and the ability of working together after the implementation of the DLGs online. As explained by T1, it was difficult to give the other regular lessons apart from the DLGs online, and one main difficulty was problems in turn taking.

“We wouldn't have done it [group lessons], because I saw difficulty when I proposed working together for example in Sciences class (...) they would get lost. They would intervene, one and another one, interventions would overlap.” (T1 interview)

However, this did not happen while in the DLGs. The interactive environment created in the DLGs on-site facilitated its transfer to the online space. Following the principles of the DLG, i.e., take turns, listen to others' interventions, and respect others' opinions, made students' attitudes different, in relation to improvements in interaction patterns. Being able to share one's thoughts in an egalitarian way enabled students to share and listen to their peers' thoughts. In this regard, T1 saw a great improvement in areas such as attention, concentration, listening to each other, and turn taking. In fact, T1 stressed she observed a personal growth and maturity improvement in her students in the DLGs.

“Simply in attention, I have found it very important. I believe interaction has been super important, the attention, concentration toward the book, the listening and respect they had when waiting for the other ones to finish their intervention and start speaking. Some of them even raised their hands (...) And I think giving the turn and waiting to be given the turn has been really adult-like, really mature. They don't do that when we are in class. As I have told you, a problem I see in the workshops was the overlapping of ideas and, however, here [in the DLGs] it has been like…” (T1 interview)

The other teacher (T2) also appreciated the level of attention of the students toward the book. According to her, it was noticeable that students had read the book and had focused on the things that were the most interesting for them when doing it in order to bring the ideas to the gathering.

“But they have read it, the other day S5 constantly with “Dulcinea, Dulcinea”, with his focus, you see each one has focused on their interest. (...) You would explain the chapter to them, and what a level of attention they had, I have found it wonderful.” (T2 interview).

Also, the reading content has been a key aspect to trigger students' cognitive progress. Teachers report that the DLGs have fostered cognitive development by discussing many topics that arose in the debates and that would have not happened without this activity. After participating in the DLGs, teachers had a clear opinion that having an intellectual disability does not impede participating in DLGs; on the contrary, although difficulties may arise, students with disabilities can benefit from this practice at different levels, even when it is implemented in the distance.

T1: I believe it can condition them, but it depends on what you want to achieve from your students (...) in our way, I think it has been useful for them to grow, mature and interiorize.
T2: Yes, yes, yes. Concepts, group relationships, we have talked about many topics that may have not aroused if we weren't doing this.
T1: I think it helps them grow, obviously. (Interview)

### Fostering Curricular Learning Despite School Closure

Participants of the study also reported curricular learning as one of the main outcomes of the DLGs online. Learning new vocabulary, improvement in comprehension, and doing so while reading such an important book—a classical book—were mentioned. Thus, it can be seen that DLGs in confinement did not impede developments in the curricular level, but even fostered new learning. As an example, T1 regarded the DLGs not just as an activity but as a subject itself, due to the wide range of contents the students learnt:

“It has been almost like a subject, we would say. In the end, it has been a subject in language. Yes, because we have worked on all the aspects of language, the comprehension, synonyms, vocabulary… (T1 interview)”

Participant students also agreed on this aspect. For instance, in the focus group, a teacher explained that students enjoyed learning new words and a different language while reading, which was confirmed by a student:

T1: I think it has been useful, we have learnt a lot of vocabulary, right? Old vocabulary, that way of speaking of chivalrous novels (...) What do you think about it?
S2: Good.
T1: S2 has enjoyed seeing another kind of language that he usually likes, he has enjoyed learning synonyms, new words, am I wrong?
S2: No. (Focus group)

This enjoyment of learning was accompanied by an improvement in the literacy level of the students, according to the teachers' perception. Although they have not been able to quantify it because the lockdown lasted the entire school year, T1 perceives such improvement as a reality:

“We can't evaluate it because it hasn't been face-to-face, and it has been the third trimester. (...) Obviously, most likely it has been positive in terms of reading, looking for the vocabulary, having a theme in common to be able to present it in class (...) The assessment is positive.” (T1 interview)

The observations of the three online DLG sessions show that students have understood the text. Students have shown to remember what they read in previous chapters and the main argument of the story. An example of this is in the third observed session, where they remember together what Quixote has done for his beloved Dulcinea.

T1: What things has Don Quixote done for Dulcinea? Who has he fought against?
S2: Against giants.
(...)
T1: And with the sheep, what did he think the sheep were? Warriors?
S4: Warriors. (at the same time)
T1: And what were they really?
S3: Sheep. (Observation session 3)

Finally, one of the aspects that teachers valued most was the fact of reading universally highly valued literature. Reading *Don Quixote* was mentioned in the interviews as a great achievement:

“And we have read *Don Quixote*, that is an important nuance.” (T2 interview)

In this vein, the reading of such a book was not only an element that helped them learn more language but also was regarded as an element that could help these students with special educational needs integrate in the society by having access to culturally relevant knowledge. Therefore, DLGs can not only facilitate students to improve their functional learning, for instance regarding language, but also enhance their cultural knowledge that can be shared in conversations with others:

“No, and they live in another reality as what happened with the stuff about Egypt, right? That sometimes those realities are in your own neighborhood, or news from the newspapers, and that these kids need functional learning and this kind of thing, something that can help them integrate. And in a certain moment, [saying] ‘I have read Don Quixote,’ well, yes. It is important.” (T2 interview)

### Enhancement in Socialization: Maintenance of Relationships and Reinforcement of the Group

In relation to the improvement of the relationships inside the group, both teachers and students emphasized the maintenance of relationships and a reinforcement of friendship even during a lockdown period due to the DLGs.

Teachers expressed that they perceived a shift from individualist attitudes to a greater union of the group. It is noteworthy that this change happened precisely when physical distancing was compulsory. However, they also acknowledge that the fact of not seeing students face-to-face makes it difficult for them to know the extent of this change.

T1: I tell you the same, which I would have liked to see it here, for example, in class. But I think it has enabled them to be much more in harmony, I think the relationships have improved a little bit. Sharing a space, a time, and a common topic, I think so. (...) There hasn't been so much individualism, at least in that moment, but whether it has made a greater union? I think everything has its positive part.
T2: I have seen that union has been made in the group. (interview)

In this regard, the data also points out that the students were looking forward to the moment of the DLG. This was something that was said to happen when DLGs were done in face-to-face class, but it was maintained even when the DLGs were carried out online. It was seen that the thrill the students had for the gathering was not diminished because of school closure and that they kept being motivated.

T1: But, in addition, notice that on Mondays they were already saying: ‘we have the gathering tomorrow.’ That for them is like, I think it is something very valuable, right?
T2: Because [researcher] came.
T1: No, but I mean in the online sessions, eh. It has been like: ‘oh, look at them, how motivated.’ (Interview)

In the words of the teachers, the fact that their students have not refused to do the DLGs is an indicator of liking them, as they tend to avoid the activities they do not like.

“They have not refused to do the activity, which means they have liked it. The thing they don't like, they say it clearly: ‘no, no, we don't want to do that’” (T2 interview).

From the point of view of students, the feelings they said to have before starting each DLG are in line with what teachers said, that they were excited about doing the activity. When asked about the feelings they had before entering the videoconference of the DMG, three students answered:

S3: Excited!
S1: Nervous.
S2: Happy. (Focus group)

The students added that one of the main things that the DLGs have provided them with has been friendship, due to the time spent together in the videoconference. For some participants, friendship has been the main learning from the DLG online sessions, above other instrumental learning such as learning new words, and it has been very important for them.

Researcher: What have you learnt the most? T1 has said vocabulary, can you think of anything else? S1? S2?
S2: Friendship.
Researcher: How important, I think when we connected together there was more friendship, is that what you think?
S2: Yes.
(...)
Researcher: What do you think has made friendship grow? Being together, talking about the same topic, sharing opinions…
S2: The time. (Focus group)

Maintaining the relationship during the lockdown through the DLGs has also translated into making plans together for when the confinement situation would be over. It is significant, therefore, that the group united during the lockdown, which teachers attributed to the DLGs. For example, the whole group decided to go to have lunch together when the situation was over. This can be interpreted as an indicator of the good relationships, enjoyment, and the desire for being together.

“It has maintained us together and it has served, you know, to say that after the work we have done we deserve a lunch. Because I think that idea has aroused thanks to all this work we have done, because it has unified us much more. I think so. The relationship between us has improved. Working together has augmented cohesion.” (T1 focus group)

## Discussion and Conclusions

The pandemic caused by COVID-19 and the measures taken to tackle its spread, such as the lockdown and school closure, have created new challenges to which teachers, families, and students have had to adapt to, especially in non-university education, which is designed to be done face-to-face and not virtually, making it difficult to carry out during the confinement (Cabrera et al., 2020). Nevertheless, vulnerable populations such as students with special needs and their families have suffered more severe consequences (Hole and Stainton, 2020).

In this context, the case study conducted shows the impact of an evidence-based strategy such as DLGs to overcome those difficulties by contributing to a quality distant learning for students with disabilities. The positive impact interactive learning environments have on students with special needs, in relation to both better academic outcomes and social inclusion, has already been studied (García-Carrión et al., 2018), and it has been shown that interactive environments can also be recreated in special schools. This study adds to that topic by presenting evidence that shows interactive learning contexts can be carried out in an online environment with students with SEN. Simultaneously, these students benefit from an interactive learning environment where interactions between families, students, and teachers have enabled curricular learning, an improvement in areas of development and behavioral aspects such as attention and turn-taking, and improvement in the relationships between the members of the class.

In order to transfer the DLGs to an online space, some key strategies were useful to leave no student behind. These strategies included audio adaptation, using pictures, explanations, and gestures that facilitated the understanding of the text and the participation of all students. Previous research had already shown that some material adaptations can support the transference of interactive learning environments to the special education context (García-Carrión et al., 2018). Our study adds new evidence showing this possibility also in distance education. This occurs when a transformative view of the situation is used, which focuses less on the difficulties (in this case related to students' special needs and to the physical separation) and more on students' capacities and the way the available resources (such as technology) can be used to maximize them.

Families have played a key role for ensuring that these students could participate in interactive spaces. Families' involvement in their children's education has been pointed out in scientific literature as a very important factor to improve both academic outcomes (Harris and Goodall, 2008) and relationships (de Botton et al., 2014; Girbés-Peco et al., 2020). Besides, in an online space, families' collaboration has also been said to guarantee the effectiveness of online teaching with students with SEN (Parmigiani et al., 2020). Our study also supports this evidence.

The case study shows that online DLGs have fostered curricular learning in confinement, especially learning of new vocabulary and improvement of comprehension, challenging the low expectations students with SEN often face in relation with their academic outcomes (Molina Roldán, 2015). Other studies on the DLGs online have also shown improvements in language and sentence construction (Ruiz-Eugenio et al., 2020), and our study points in the same direction when students with special needs are the participants. In addition, the reading of a universally valued novel such as *Don Quixote* with students with special needs was regarded by the teachers as an element of social inclusion. Thus, the online DLGs created a learning environment where the special needs of students did not impede them to enjoy this masterpiece.

An improvement in social relations within the class was also reported as a result of the implementation of online DLGs. This is consistent with other studies that have revealed interactive learning environments to improve both academic improvement (Valero et al., 2018) and friendship relationships among students (León-Jiménez et al., 2020). It is significant in our study that the relationships between students and teachers were not only maintained but also became stronger while doing online DLGs.

Overall, online DLGs have been crucial to promote interactions during the lockdown and as an alternative to individualized classes. Additionally, it is worth highlighting that the benefits reported have been observed after only 6 months implementing DLGs (either face-to-face or online). Taking into account the benefits interactive learning has proven to have on students with special needs (Molina Roldán, 2015; García-Carrión et al., 2018; Fernandez-Villardon et al., 2020), having the possibility to transfer DLGs to the online modality as a way to maintain these interactions even in lockdown is important, on the one hand because school closure due to COVID-19 continues today in some contexts and, on the other hand, because new confinements could be applied in other contexts while the sanitary emergency continues. Our results open new opportunities for other schools teaching students with special needs to keep this environment of interaction, learning, and group cohesion.

Some limitations must be noted. First, the size of the benefits of the online DLGs has not been measured, as the remaining school year was done fully telematically. Therefore, the improvements reported are based on perceptions of the teachers, students, and observations, but other records of academic improvement could not be analyzed. Second, our data were collected after a period of 6 months implementing the DLGs; therefore, it is unknown if the benefits would sustain after a longer implementation period. Thus, future research could address the stability and maintenance of the effects of online DLGs, as well as the measure of the size of those improvements.

## Data Availability Statement

The raw data supporting the conclusions of this article will be made available by the authors, without undue reservation.

## Ethics Statement

The studies involving human participants were reviewed and approved by Ethics Committee of the Community of Research on Excellence for All (CREA). Written informed consent to participate in this study was provided by the participants' legal guardian/next of kin.

## Author Contributions

SR-P contributed to the conception and design of the study. AL and GÁ-G organized the database. AL and SR-P wrote the first draft of the manuscript. GÁ-G, LF-M, and SR-P revised and edited the manuscript. All authors contributed to manuscript revision, read, and approved the submitted version.

## Conflict of Interest

The authors declare that the research was conducted in the absence of any commercial or financial relationships that could be construed as a potential conflict of interest.
